# In Their Own Words: How Family Carers of People with Dementia Understand Resilience

**DOI:** 10.3390/bs7030057

**Published:** 2017-08-21

**Authors:** Siobhan T. O’Dwyer, Wendy Moyle, Tara Taylor, Jennifer Creese, Melanie Zimmer-Gembeck

**Affiliations:** 1 Medical School, University of Exeter, Exeter EX1 SLU, UK; 2 Menzies Health Institute Queensland, Griffith University, Nathan, QLD 4111, Australia; w.moyle@griffith.edu.au (W.M.); tara.louise.taylor@gmail.com (T.T.); jenny.l.creese@gmail.com (J.C.); m.zimmer-gembeck@griffith.edu.au (M.Z.-G.); 3 School of Nursing and Midwifery, Griffith University, Nathan, QLD 4111, Australia; 4 School of Applied Psychology, Griffith University, Nathan, QLD 4111, Australia

**Keywords:** dementia, caregivers, resilience, acceptance, adversity

## Abstract

There is a growing body of research on resilience in family carers of people with dementia, but carers’ voices are noticeably absent from it. The aim of this study was to explore carers’ definitions of resilience and their opinions on the factors associated with resilience. Twenty-one in-depth interviews were conducted in Australia with people who were currently, or had previously been, caring for a family member with dementia. Transcripts were analysed thematically and three themes emerged: the presence of resilience, the path to resilience, and characteristics of the resilient carer. Although carers struggled to define resilience, the vast majority considered themselves resilient. Carers identified a range of traits, values, environments, resources, and behaviours associated with resilience, but there was no consensus on the relative importance or causal nature of these factors. Carers also considered resilience to be domain- and context-specific, but did not agree on whether resilience was a trait or a process. These findings highlight both the importance of including carers’ voices in resilience research and the limitations of the extant literature. There is much to be done to develop a field of carer resilience research that is theoretically sound, methodologically rigorous, and reflects the lived experience of carers. A model is provided to prompt future research.

## 1. Introduction

More than 46 million people worldwide are currently living with dementia and the majority are cared for by a spouse, friend, or adult child [1]. Already people aged over 65 account for 34% of family carers and, as the population ages, caring (and particularly caring for a person with dementia) is expected to become an increasingly common feature of later life. Although families provide the ‘cornerstone’ of dementia care [2], the negative consequences of being a family carer are well documented. There is an extensive body of literature on the negative physical and psychological outcomes of caring for a person with dementia [3,4], including a growing body of research on suicidal and homicidal ideation [5,6]. Less well understood, however, is the way in which some carers are able to avoid or mitigate negative outcomes and even thrive as a result of the caring experience [7,8]. Researchers seeking to understand the positive outcomes of caring for a person with dementia have turned to the construct of resilience. 

Resilience research began with studies of children who had experienced deprivation or trauma [9,10,11,12,13] and has recently expanded to include older adults and carers [14,15,16,17,18,19,20,21]. Across this literature, however, there is considerable variation in how resilience is defined and measured [13,22,23,24]. Definitions include: the achievement of developmental milestones despite adversity; better than expected outcomes in the face of adversity; the maintenance of previous levels of functioning despite adversity; a return to previous levels of functioning after adversity; and the surpassing of previous levels of functioning (‘thriving’ or ‘flourishing’) [10,12,15,25,26,27,28]. The definition of adversity also varies across studies, as does the timeframe, domains, and contexts in which both resilience and adversity are examined. Furthermore, while some scholars consider resilience to be a personality trait, others consider it a dynamic process, and although some consider resilience to be either present or absent, others propose a continuum from vulnerability to resilience [10,15,26]. Resilience and adversity have also been measured quantitatively and qualitatively, in cross-sectional studies and over time [24]. Despite differences in definition and measurement across studies, however, there is broad consensus that the factors influencing resilience span biological, psychological, social, and environmental domains. 

The problems of definition and measurement also plague the literature on resilience in people caring for family members with dementia [25,29,30,31,32,33,34]. The relative infancy of the field also means there is a lack of rigorous, systematic research on the factors that influence resilience in this population. Also noticeably absent from this literature are the voices of carers themselves. To our knowledge, only three resilience studies have interviewed people caring for family members with dementia [25,35,36], but neither asked carers whether they considered themselves resilient. Similarly, although Joling and colleagues [37] included carers on an expert panel in a Delphi study that sought to define resilience in people caring for family members with dementia, they were only asked about resilience generally, not about their own resilience or the factors that contributed to it. 

Asking family carers of people with dementia about their own definitions, perceptions, and experiences of resilience might provide important insights into the process and experience of resilience in this population. As Luthar and colleagues [13] note “the meaning of a particular adverse event to the individual experiencing it can differ substantially from that of the resilience researcher…Some individuals may see themselves as being relatively well off, even though scientists may define their life circumstances as being highly stressful” (p. 550). Further, as Wild and colleagues [22] observe, “people’s definitions tend to be more multidimensional, nuanced, contextualised, and attentive to balance and tension than the rather more narrowly focused definitions often used by researchers” (p. 153).

The aim of this study was to examine carers’ definitions of resilience and the factors they think contribute to their resilience during caregiving. As the research was exploratory, a descriptive qualitative approach was taken.

## 2. Materials and Methods

### 2.1. Participants

Sixty-one Australian carers from a large, cross-sectional survey of carer health and wellbeing [6] gave consent to be contacted for (and subsequently agreed to participate in) a follow-up interview. Participants in the original study were a convenience sample, recruited via newspaper advertising, promotion at community events and conferences, direct invitations via advocacy organisations and care providers, social media, radio interviews, and word of mouth. Participants were eligible for the original study if they could read English and identified themselves as the primary provider of care for a family member, friend, or spouse with dementia who was either living in the community (home care, HC), was in long-term care (in care, IC), or had died within the past two years (bereaved, BD). The study was framed as a broad exploration of the caregiving experience and prospective participants were made aware that the interview would contain questions about suicide, homicide, resilience, and support.

To ensure the inclusion of a diverse range of participants and experiences, maximum variation sampling (MVS) [38] was used to select carers for interview. Gender, care type (HC, IC, BD), caring relationship (spouse, parent, sibling), location (urban, rural/remote), and cultural diversity (migrant; lesbian, gay, bisexual, or transgender (LGBT); indigenous) were the dimensions of variation used to select participants. On the basis of these factors, 21 participants were selected for interview.

### 2.2. Procedure

An interview protocol, including a semi-structured interview guide, was developed by SOD and WM, in consultation with a registered psychologist. To establish rapport, participants were asked to provide a brief overview of their caring experience at the beginning of the interview. The questions then focused on resilience, support, and thoughts of suicide and homicide. For the purpose of this study, the analysis focused only on responses to questions about resilience. 

The interviews were conducted individually, by telephone, and by a registered psychologist (TT). All interviews were digitally recorded and transcribed verbatim. The interviews ranged in length from 38 to 106 min.

The study was conducted in accordance with the Declaration of Helsinki, the University Human Research Ethics Committee approved the study (reference number NRS/20/11/HREC), and all participants provided signed, informed consent. Due to the inclusion of questions on suicide and homicide, a number of risk assessment, risk response, and debriefing procedures were employed. A detailed description of these is available in [6]. 

### 2.3. Analysis

The thematic analysis followed the steps identified by Braun and Clarke [39]: data familiarisation through reading and re-reading the transcripts; generating initial codes in a systematic fashion across the whole data set; identifying themes within identified codes; reviewing themes for internal and external validity; and defining and naming themes. One member of the research team (JC) who had not been involved in developing the interview questions or conducting the interviews, conducted the initial analysis. After the initial analysis was complete, the codes and themes were reviewed and revised by a second researcher (SOD). The revised analysis was then discussed with a third researcher (WM), with discussion continuing between the researchers until consensus was reached.

## 3. Results

### 3.1. Participants

The 21 participants ranged in age from 37 to 89 years (M = 67; SD = 14). Ten were caring at home, four had placed the person with dementia into long-term care, and seven were bereaved. The majority (*n* = 16) were spousal carers and five lived in rural/remote areas. Table 1 contains a description of each participant.

### 3.2. Themes

Three themes were identified in the data: the presence of resilience, the path to resilience, and characteristics of the resilient carer. These themes are discussed below, with representative data extracts provided to illustrate the themes. Across these themes there were no obvious differences between those caring at home, those supporting a person with dementia in long-term care, and those who had been recently bereaved, so the findings have been reported for the sample as a whole. Before presenting the themes, however, it is important to comment on the definition of resilience that guided these responses.

Consistent with the aim of this study, the interview guide originally included a question asking participants for their definition of resilience. The first four participants all struggled to respond to this question and none were able to provide a definition. When the interviewer provided a definition, however, all four offered clear, considered opinions on their own resilience and that of others. Consequently, the remaining participants were not asked to define resilience. Informed by the extant literature, the definition provided in all interviews was: the ability to bounce back after a challenging situation; being able to recover from, resist, or adapt to the physical and psychological demands of caring.

### 3.3. The Presence of Resilience

Nineteen of the 21 carers described themselves as resilient. These carers said they were resilient because they had endured many challenges, persevered despite setbacks, ‘bounced back’ from difficult experiences, and grown as a result of those experiences. It was clear, however, that the majority of these carers had never reflected on their experiences in this way before; that the demands of caring for a person with dementia had afforded them few, if any, opportunities to pause and consider their achievements.

I mean I’m sure I must be because I’ve gone through a lot of difficult things.

(Participant 15)

Well I suppose I am, yeah. I’ve come through [caring for my son] and I’m still going [caring for my husband].

(Participant 9)

I’d say so. We’ve had a great life doing all sorts of things, but we’ve had our tough times too. We’ve always bounced back.

(Participant 10)

I’ve grown out of this [caring experience].

(Participant 13)

Despite initially saying they were resilient, two male carers went on to question the extent and value of their resilience. One said: “*It’s survival rather than resilience*” (Participant 11). The other said: “*I suppose if resilience consists of getting knocked down and keep on getting up, there might even be an element of stupidity in that*” (Participant 2). For these carers, the development of resilience was not necessarily a positive experience, but rather one they would happily have forgone had the alternative been not ‘getting knocked down’. 

Some carers also said that there were, or would eventually be, limits to their resilience. For these carers, resilience was not ‘all or nothing’, but rather something that was mutable and dependent upon a range of personal, social, and environmental factors. Although these carers were demographically diverse, they shared high levels of insight into their own emotional processes and an appreciation of the unpredictable nature of both caring and life.

I don’t think I’ll always be resilient. I think you can be rocked. [Resilience] can be destabilised by a number of things … Life’s good and I would call myself very resilient, but last year I was a mess again … So I think there are things that can destabilise you. I don’t take it for granted.

(Participant 19)

When I got home, all I did was lie on the couch. I kicked off my shoes and cried my eyes out for hours and hours … The next day I got horribly sick. I think my whole body crashed. I think I was sick for about three days … I think I kept my strength up when [my husband] was around, but when I put him in the facility everything just crashed on me.

(Participant 18)

### 3.4. The Path to Resilience

There was considerable diversity in how carers thought they and/or others became resilient. Three carers, for example, said their resilience was innate. For these carers, resilience was an enduring personality trait that could not be acquired, but rather was present (or absent) from birth.

I think I was born with it.

(Participant 5)

I think I’ve always had it.

(Participant 18)

Six carers said that although their resilience was innate, it was possible for others to learn to be resilient. Being ‘born resilient’, however, was clearly the more desirable option.

I tend to be resilient by nature. [My friend who is also a carer] learnt to be. We’re both sides of that scale.

(Participant 12)

I suppose you could probably learn to be resilient. It would probably be a hard road to hoe … [It’s] more something you’re born with rather than something yeah, you know, I think you could learn to become resilient, but it would be a hard job, I think.

(Participant 10)

I think it’s something you could either be born with or it’s the way you were brought up … I believe you can [learn to be resilient] but I think it, to learn it, I think takes a while … I don’t think you could learn to become a resilient carer just by attending a lecture … I think you’ve got to have ‘hands on’ experience as well.

(Participant 13)

Eleven carers said that their resilience had been acquired, either through upbringing or during adulthood. For these carers, the development of resilience was an experiential, cumulative process and their comments reflected a sense of pride in having survived and thrived despite difficult circumstances.

Well, you’re not born with it, it is something that you have to earn.

(Participant 17)

I think my resilience was built up by having no Mum and living with my Dad … I lost Mum when I was four and I learned the hard way.

(Participant 3)

I think it’s my own inner strength that I built up over the years and the knocks that we’ve had. That made me a stronger person.

(Participant 4)

Approximately half the carers also said that regardless of whether resilience was innate or acquired, it was a necessity. Faced with the relentless demands of dementia care, these carers felt that resilience was their only choice. For them, resilience was about accepting the situation as it was and being resolved to carry on.

I think perhaps with dementia you have to become resilient or you won’t survive. You’ll either commit suicide or harm the person that you’re with or collapse in a heap.

(Participant 6)

I can’t just curl up in a ball … I can’t take to my bed or anything for a couple of days, because he needs looking after … So I suppose that’s why I’m resilient. Because I’ve got to look after him.

(Participant 9)

You’ve got to be. Yes, I’ve got to be. Maybe not naturally or normally, but no, I must be. I must be prepared to stand up.

(Participant 11)

Although a few carers identified luck as a part of becoming resilient, the circumstances they described were clearly the consequence of higher socioeconomic status. In particular, their social and financial circumstances ensured better access to the practical and emotional supports that facilitated their resilience.

I was extremely lucky having the support I got. I really think that not many people have the support I had.

(Participant 5)

We were lucky that Mum and Dad were self-funded retirees, that we could afford to put [Mum] in the [aged care facility].

(Participant 7)

Similarly, four female carers—one the daughter of Greek migrants—said that they were just ‘the resilient one’ in their family or relationship. Their comments, however, clearly reflect the influence of gender and cultural norms on both experiences of caring and expectations about resilience.

If anything happens the family ring me and I sort of straighten things out … Organise Dad’s funeral, organise everything … yeah, that’s me.

(Participant 8)

I’ve been caring all my life. I was the child of migrants and the youngest child. That was just my role.

(Participant 15)

### 3.5. Characteristics of a Resilient Carer 

Carers identified a range of traits, behaviours, and values they felt characterised a resilient carer, as well as a range of environmental factors and resources they felt were available to resilient carers. The traits included being proactive, achievement-oriented, determined, self-aware, self-reliant, fearless, patient, flexible, creative, and resourceful. The values included love, commitment, and care, while the environmental factors included practical and/or emotional support, access to respite care, and outlets or hobbies. The resources included information about dementia and support services, money, and good health, while the behaviours included acceptance, self-care, delegation, preparation, reciprocation, seeking information and support, developing routines and strategies, learning from others, expressing emotions, building skills, reflecting on experiences, persevering, moving forward without forgetting the bad times, walking away or letting go, and keeping things in perspective. 

Within these comments, however, the relative importance of the various characteristics was not clear, nor was there any consistency in whether carers viewed resilience as a necessary precursor to, or the positive consequence of, these characteristics. The comments did not align in any meaningful way with carers’ perspectives on their own resilience (acquired vs. innate) and there was no discernible pattern in the data regarding gender, stage of care, or relationship to the person with dementia. 

While a few carers said that a lack of these characteristics (particularly resources and a supportive environment) made their caring experience more challenging (e.g., “*A lot of people don’t know help’s available. I didn’t at the time. I just didn’t know*”—Participant 5; “*I could, in a way, kind of cope with the fact that she was diminishing. As bad as that is, I could accept that. But getting people on board to help was incredibly difficult*”—Participant 15), all cited at least one characteristic which they possessed that made them resilient, with 19 of the 21 citing characteristics they possessed across three or more of the categories (traits, behaviours, environment, resources, values).

## 4. Discussion

As the population ages and caring for a family member with dementia becomes a common component of later life, understanding and promoting the positive aspects of caring will become increasingly important [8]. To our knowledge, this is the first study to ask family carers of people with dementia to define resilience and to consider their own resilience and the factors that contribute to it. Carers had such difficulty defining resilience, however, that the question was removed from the interview protocol. This suggests that although the term ‘resilience’ may have academic merit, it is not a term that carers are using to describe their own experiences. 

Despite this, when a definition of resilience was provided, 19 of the 21 participants said they were resilient (the remaining two failed to give a clear answer to this question). This is a marked difference from Donellan et al.’s research [25], in which the researchers classified only eight of their 20 carers as resilient, and Joling et al.’s research [34], in which only 35–43% of carers were identified as resilient. This discrepancy highlights the importance of seeking carers’ views on resilience and supports the idea that external perceptions of adversity and resilience do not always align with lived experience [13,15,37]. The vast majority of carers defining themselves as resilient is also consistent with Masten’s [9] assertion that resilience is “ordinary magic” (p. 227), a natural human process of adaptation that should be considered the norm rather than the exception. 

It is also interesting to note that when explaining why they considered themselves resilient, several carers in the current study mentioned growth, a feature of resilience that was not included in the definition we provided. There is considerable diversity in the literature regarding whether definitions of resilience should include growth or thriving as a necessary component, or whether a return to, or maintenance of, previous levels of functioning is sufficient. The current study suggests that for people caring for family members with dementia, growth may be an important marker of resilience. These carers also considered acceptance of difficult circumstances to be an important aspect of resilience. This aligns with O’Rourke et al.’s finding that considering change to be a normal part of life is central to resilience [30] and lends support to the growing focus on acceptance- and mindfulness-based therapies for family carers of people with dementia [7,40,41]. 

Carers in the current study also considered resilience to be both context and domain specific. For them, resilience in one situation was not a guarantee of resilience in another and resilience now was not a guarantee of resilience in the future. Although this is consistent with much of the literature on childhood resilience, it has not been reflected in the measurement, operationalization, or description of resilience in the literature on family carers of people with dementia, e.g., [25,29,30,31,32,35,37], in which carers have frequently been labelled as either resilient or not. It was clear that carers in the current study also considered it possible to be both resilient and distressed. This stands in contrast to the work of Donellan et al. [25]—who included a lack of distress as a criterion for resilience—but is consistent with the broader resilience literature in which highly-resilient children have also been shown to experience high levels of distress [11]. Labelling family carers of people with dementia as either ‘resilient’ or ‘not resilient’ risks framing the ‘non-resilient’ as beyond help and the ‘resilient’ as not in need of help [12,13,22]. Similarly, mandating an absence of distress frames resilience as something to be achieved, rather than a dynamic interplay of myriad factors; an oversimplification which is unlikely to facilitate the type of complex interventions required to enhance resilience [11,42]. 

Although the majority of carers considered themselves resilient, there was a lack of consensus in their views of resilience as a trait or process. Regardless of whether they thought resilience was a trait or a process, however, the majority of carers spoke in terms of skills and resources they had (or had acquired) prior to becoming carers. This suggests that when considering resilience in family carers of people with dementia, the context within which the adversity occurs is important [22]. It also suggests that positive or negative outcomes of previous adversity might influence experiences and perceptions of future adversity, consistent with the idea of resilience as a cyclical process [26]. 

Carers also varied in the factors they thought were associated with resilience and whether they considered these factors to be causes or consequences of resilience. Although their responses support the notion that a broad range of biological, psychological, social, and environmental factors can facilitate or inhibit resilience in carers [18,19,20,22,34], the lack of coherence presents both a challenge and an opportunity for future research and practice. Until the relative importance and causal influence of these factors are identified, it will be difficult to develop effective interventions to enhance resilience in people caring for family members with dementia [42]. Longitudinal research and life review methods (in which participants identify and reflect upon major life events; [15,21], however, might help to tease out the role of different factors. 

Although this study provides an important insight into carers’ perceptions of resilience, it is not without limitations. Firstly, carers were not asked to define adversity. Their narratives do suggest, however, that care-related adversity can be acute and/or cumulative and varies according to the individual. To date, research on resilience in family carers of people with dementia has varied in how it has operationalized adversity, with some studies assuming that all dementia caring is an adverse experience, e.g., [25,37], others using measures of burden, perceived stress, or care demands as proxies for adversity, e.g., [29,31,32,34], and none examining the outcomes of specific incidences of adversity, cumulative adversity, or changes in resilience over time. Caring for a person with dementia is not an homogeneous experience and both adversity and responses to it may vary between carers and over time. Further, high-functioning carers may not be resilient, but simply not have experienced high levels of adversity [13]. Longitudinal research, as well as qualitative research examining carers’ definitions and perceptions of adversity, would provide greater insight into the relationship between adversity and resilience in family carers of people with dementia. 

Secondly, in-depth qualitative methods do not allow for an exploration of the biological factors associated with resilience. Some compelling research has recently been conducted on cortisol levels in people caring for family members with dementia [43,44] and incorporating these methods into resilience research would help to further understand the biological mechanisms that might facilitate or inhibit resilience in this population. 

Thirdly, this research was conducted with a small sample of Australian carers. Although MVS was used to increase diversity in the sample, the influence of demographic factors on perceptions and experiences of resilience could not be determined. The data did, however, hint at the importance of gender and socioeconomic status, as well as culture and ethnicity, and larger studies would help to explore the influence of these factors. Similarly, despite efforts to ensure a diverse sample, it is also possible that carers who were more resilient were more willing or able to participate in the research. 

Finally, this study focused specifically on people caring for family members with dementia. People caring for family members with physical disabilities, chronic illness, or mental illness might have different opinions and experiences of resilience [45]. Research that compares and contrasts the views of different carer groups would help to further clarify the aspects of resilience that are specific to caring, those that are specific to dementia, and those that are universal. 

**Figure 1:**
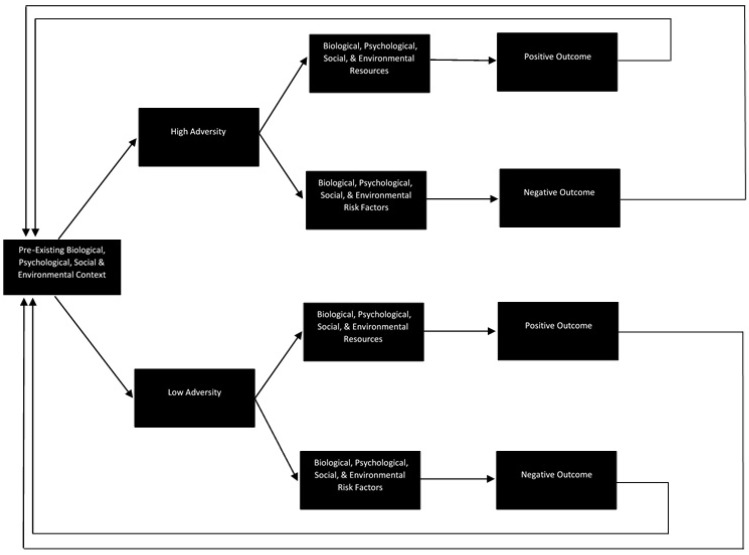
A proposed model of resilience in family carers of people with dementia.

In attempting to situate the current findings in the extant literature, it has become clear that there is much to be done to develop a field of research on resilience in family carers that is theoretically sound, methodologically rigorous, aligned with the broader resilience literature, and reflects the lived experience of carers. Although Windle and Bennett [18] have proposed a framework that has guided some of the more recent dementia carer resilience studies, the framework fails to account for many of the issues identified here. The model proposed in Figure 1, however, may go some way toward addressing them. Consistent with the childhood resilience literature and informed by the experiences of carers in this study, the model conceptualises resilience as a cyclical process; accounts for the biological, psychological, social, and environmental context in which adversity occurs; reflects the potential for adversity to be both acute and cumulative; and does not assume that all carers experience adversity or that that adversity is equal in its intensity. Due to a lack of clarity in the extant literature, and consistent with the breadth of responses provided by carers in this study, the model does not define adversity, positive/negative outcomes, or the factors that might facilitate or inhibit resilience (framed in the model as ‘risks’ and ‘resources’). A wide range of factors could be considered to reflect adversity, positive outcomes, negative outcomes, risks, and resources, and what constitutes risk, adversity, or a negative outcome in one context or for one carer may not be relevant or could even constitute a resource or positive outcome in another context or for another carer. Thus, rather than prescribing or predetermining the role of specific factors (particularly the ‘usual suspects’ like burden and strain), the model provides a framework within which these concepts (and the relationships between them) might be defined, operationalized, and tested in future research. The model, therefore, is a theoretical offering intended to stimulate discussion, prompt empirical research, and encourage a more considered approach to the study of resilience in family carers.

## 5. Conclusions

Resilience is an emerging topic in the literature on people caring for family members with dementia, but much remains to be understood. This research highlights the importance of including carers’ voices in resilience research. Although the majority of carers in this study considered themselves resilient, they varied in their views of the factors associated with resilience. A theoretical model of resilience that takes into account context, variations in adversity, and the cyclical nature of resilience may provide a useful framework for future carer resilience research.

## Figures and Tables

**Table 1:** Participant demographics.

Participant	Age	Gender	Care Type ^a^	Person with Dementia	Other ^b^
Participant 1	62	F	IC	Husband	
Participant 2	83	M	IC	Wife	
Participant 3	83	M	BD	Wife	
Participant 4	73	F	BD	Husband	Migrant
Participant 5	85	M	BD	Wife	Migrant
Participant 6	72	F	HC	Husband	Migrant, Rural/Remote
Participant 7	56	F	BD	Mother	
Participant 8	60	F	HC	Husband	Rural/Remote
Participant 9	69	F	BD	Husband	Migrant, Rural/Remote
Participant 10	74	M	HC	Wife	Rural/Remote
Participant 11	76	M	HC	Wife	Rural/Remote
Participant 12	59	F	IC	Mother	
Participant 13	75	M	HC	Brother	
Participant 14	89	M	BD	Wife	
Participant 15	37	F	BD	Mother	
Participant 16	72	F	HC	Husband	
Participant 17	48	F	HC	Husband	
Participant 18	60	F	IC	Husband	
Participant 19	52	F	HC	Mother-in-law	LGBT
Participant 20	64	F	HC	Father	
Participant 21	50	F	HC	Husband	

Notes: ^a^ HC: Caring at home; IC: Person with dementia in long-term care; BD: Bereaved. ^b^ LGBT: Lesbian, gay, bisexual, or transgender.
